# MYPN mutation-related cardiomyopathy presenting with diffuse subendocardial fibrofatty infiltration

**DOI:** 10.1093/ehjcr/ytag329

**Published:** 2026-05-08

**Authors:** Liqiong Yuan, Kai Yang, Minjie Lu

**Affiliations:** Department of Magnetic Resonance Imaging, Fuwai Hospital, National Center for Cardiovascular Diseases, Chinese Academy of Medical Sciences, Peking Union Medical College, Beilishi Road No. 167, Xicheng District, Beijing 100037, China; Department of Radiology, The Fifth Hospital of Wuhan, Xianzheng Road No. 122, Hanyang District, Wuhan, Hubei 430050, China; Department of Magnetic Resonance Imaging, Fuwai Hospital, National Center for Cardiovascular Diseases, Chinese Academy of Medical Sciences, Peking Union Medical College, Beilishi Road No. 167, Xicheng District, Beijing 100037, China; Department of Magnetic Resonance Imaging, Fuwai Hospital, National Center for Cardiovascular Diseases, Chinese Academy of Medical Sciences, Peking Union Medical College, Beilishi Road No. 167, Xicheng District, Beijing 100037, China; Key Laboratory of Cardiovascular Imaging (Cultivation), Chinese Academy of Medical Sciences, Beijing 100037, China

A 25-year-old man had a 6-year history of unexplained chest tightness and dyspnoea, which improved with rest. Electrocardiography showed sinus rhythm, abnormal Q waves, and ST-T changes. Multiple 24-h Holter monitoring documented frequent premature ventricular contractions (PVCs > 500/24 h). Three years earlier, due to a marked increase in PVCs (30 415/24 h) and mildly elevated N-terminal pro–B-type natriuretic peptide (194 pg/mL; normal < 150 pg/mL), radiofrequency ablation was performed. At that time, his absolute eosinophil count was 1.1 × 10^9^/L (normal 0.02–0.52 × 10^9^/L). Echocardiography and coronary computed tomography angiography revealed left ventricular (LV) enlargement, abnormal wall motion, and extensive subendocardial fat infiltration (*Panel A*), with no coronary stenosis. Cardiac magnetic resonance (CMR) showed an enlarged LV (end-diastolic diameter 63 mm), mild wall thinning (septum 5–8 mm; inferior wall 3–4 mm), and extensive subendocardial fat infiltration (*Panel B*: four-chamber cine; *Panels C* and *D*: short-axis water–fat separation), with an ejection fraction of 50%. Further CMR demonstrated extensive subendocardial fat infiltration and fibrosis (*Panels E* and *F*: four-chamber and black-blood late gadolinium enhancement), native T1 values of 963–1497 ms, and elevated extracellular volume (25%–47%) (*Panel G*: T1 map; *Panel H*: extracellular volume map). Endomyocardial biopsy confirmed vacuolar degeneration of cardiomyocytes and marked interstitial fibrosis (*Panel I*). Genetic sequencing identified a heterozygous MYPN mutation (*Panel J*). While arrhythmogenic ventricular cardiomyopathy typically involves subepicardial or mid-myocardial fibrofatty replacement, this case uniquely demonstrated extensive subendocardial fibrofatty replacement of the LV. A diagnosis of MYPN-related cardiomyopathy with diffuse subendocardial involvement was considered. For non-ischaemic cardiomyopathy with fatty infiltration, CMR water–fat separation and late gadolinium enhancement are highly sensitive for detecting fat and fibrosis, and genetic testing is recommended.

**Figure:**
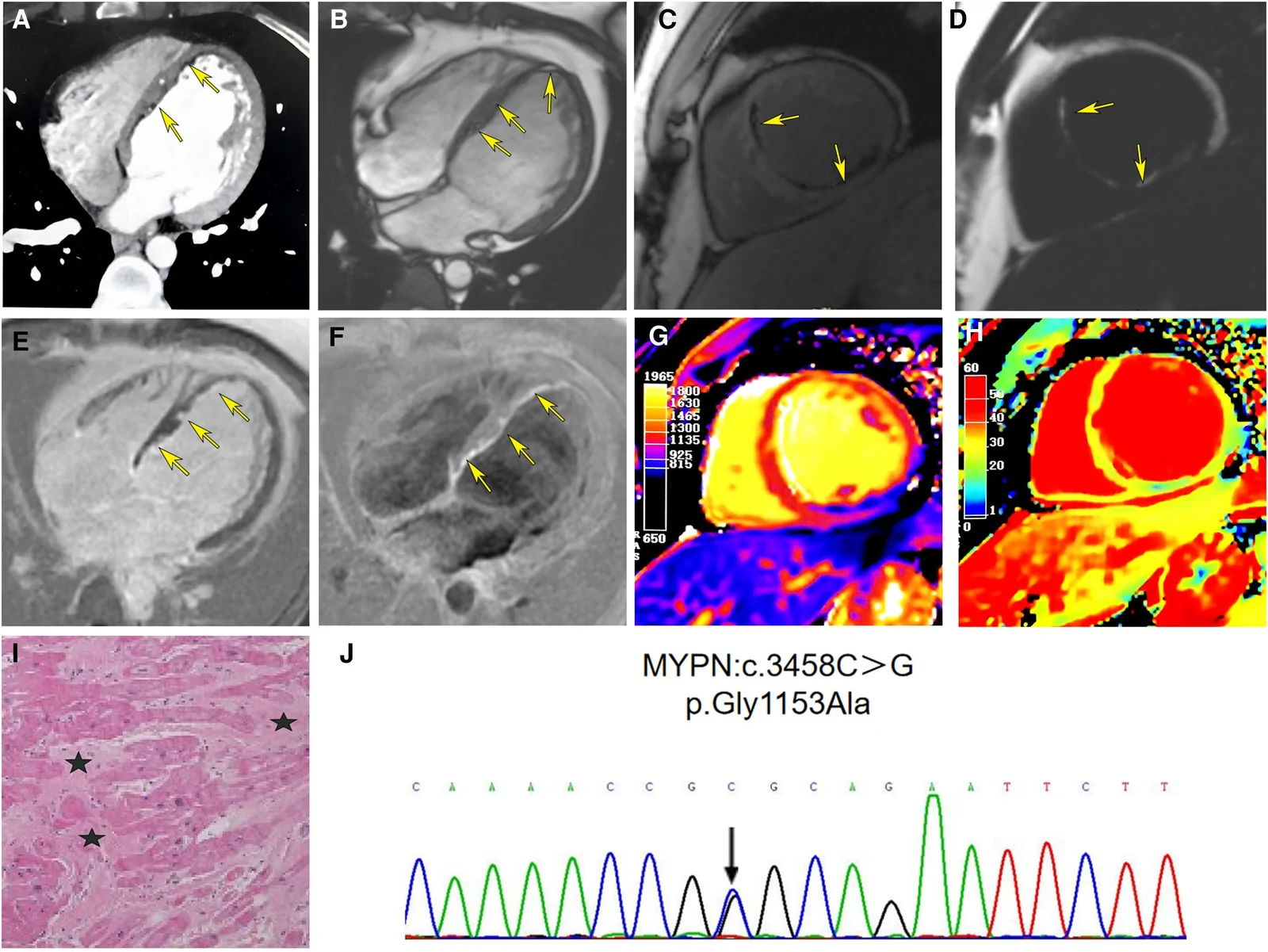


## Data Availability

The data underlying this article are available in the article.

